# Nutritional considerations to inform nutritional safety assessments of foods derived from cellular agriculture: A scoping review protocol

**DOI:** 10.1016/j.mex.2025.103658

**Published:** 2025-10-04

**Authors:** Karima Benkhedda, Matthew D. Parrott, Stephanie K. Nishi, Vincent C.H. Wong, Laura Kenney, Chao-Wu Xiao, Atiq Rehman, Subhadeep Chakrabarti, Mélanie Whiteside

**Affiliations:** a Bureau of Nutritional Sciences, Food and Nutrition Directorate, Health Products and Food Branch, Health Canada, Ottawa, Ontario, Canada; b School of Nutrition, Faculty of Community Services, Toronto Metropolitan University, Toronto, ON, Canada

**Keywords:** Cell-cultured foods, Cellular agriculture, Nutritional quality, Food safety

## Abstract

Cellular agriculture is the production of meat, milk, eggs, seafood, and other products and ingredients from cell cultures rather than from farmed animals. It is an emerging field in food innovation that has gained attention of the scientific community, food industry, and regulators around the world. This scoping review aims to summarize existing knowledge on cell-cultured food products, focusing on key aspects that impact their nutritional quality and identifying knowledge gaps in this area. Relevant publications will be identified from electronic databases and the grey literature. This review will include scientific publications (peer-reviewed journal articles; reports from regulatory, scientific, or non-governmental bodies) that report or discuss nutritional considerations concerning cell-cultured food products derived from plant or animal cells. Based on pre-determined inclusion and exclusion criteria, records from scientific databases will be screened and selected by two independent reviewers, while records from grey literature will be screened by one reviewer. Data from the included references will be extracted by one reviewer using pre-piloted extraction templates, and verified by a second reviewer. The findings will inform and strengthen Health Canada’s nutritional assessments of novel foods derived from cellular agriculture. Identified research gaps will provide opportunities for future research in this area.

## Graphical abstract

**Figure:**
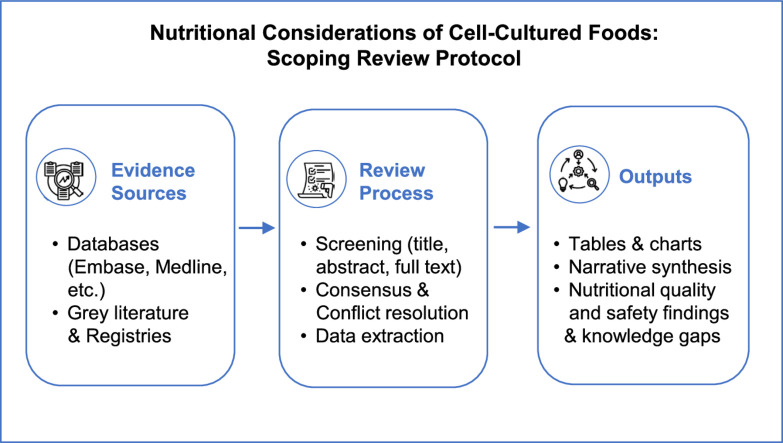

## Specifications table

**Table:** 

**Subject area**	Food Science
**More specific subject area**	Food, nutritional quality, knowledge synthesis
**Name of your protocol**	Food, nutritional quality, knowledge synthesis
**Reagents/tools**	Not applicable
**Experimental design**	Not applicable. This protocol describes a scoping review (type of knowledge synthesis).
**Trial registration**	Not applicable. This is not a trial.
**Ethics**	This work did not involve human subjects, animal experiments or data collected from social media platforms.
**Value of the Protocol**	Scientific contribution: • Systematic and comprehensive summary of existing evidence concerning cell-based foods • Identify key considerations impacting the nutritional quality of cell-based food products • Identified research gaps will provide opportunities for future research in this area.

## Background

The growing world population, globalization of the food supply, increasing demand for diverse food sources, and rapid advances in food science and technology have driven the development of new types of foods [1]. Food innovation is explored by industry as a tool to help reduce the environmental impact of food production and contribute to sustainable food systems [[2], [3], [4]]. For example, cultivated sources of animal proteins produced from cellular agriculture are viewed by some as a potential solution to meet the demand for meat and meat products [[5], [6], [7]]. However, the terminology and the types of products categorized as cell-based foods remain inconsistent in the published literature. Broadly, cellular agriculture is defined as the production of meat, milk components, eggs, fish, or seafood from cell cultures rather than from farmed animals and these products are referred to as cell-based, lab-grown, or cell-cultured foods. The safety for human consumption and the nutritional quality of these products remains to be established on a case-by-case basis [[8], [9], [10]]. In Canada, products of cellular agriculture such as those derived from cultivated animal cells are considered novel foods and require mandatory premarket safety assessment by Health Canada before they can be sold in the Canadian market [11]. The safety assessment of novel foods by Health Canada addresses several aspects including characterization of the products, toxicological, chemical, microbiological, allergenicity, and nutritional considerations. The nutritional assessment of novel foods considers the presence of essential nutrients and energy-yielding substances in appropriate quantity and quality, nutrient bioavailability, and nutrient interactions with other nutrients, food additives and natural toxicants. Consideration is also given to nutrient excesses and the effects, both positive and negative, of food processing on the nutrients and on the organoleptic properties of the food. With this insight, the objective of this scoping review is to systematically identify and summarize existing knowledge on nutritional considerations of foods derived from cellular agriculture and to highlight knowledge gaps in this area. The findings will inform the nutritional safety assessments of these foods and identify knowledge gaps to be tackled by food innovators and researchers. A preliminary search was conducted in PubMed and PROSPERO to identify any prior scoping reviews addressing this review’s research question, but no relevant records were found.

## Description of protocol

The scoping review protocol was developed to explore the available evidence on the nutritional safety of foods derived from cellular agriculture, aiming to identify key nutritional safety considerations and existing knowledge gaps. The findings are expected to inform and strengthen the development of assessment methodologies to characterize nutritional hazards and risks to human health related to cell-based foods, an area of interest for regulators worldwide.

A preliminary search for existing scoping reviews addressing the research questions of this scoping review has been conducted in PubMed and PROSPERO to identify any prior scoping reviews published or registered in this field. No records were found.

### Methods

The proposed scoping review will be conducted following the JBI methodology for scoping reviews and reported in line with the Preferred Reporting Items for Systematic Reviews and Meta-Analyses extension for Scoping Reviews (PRISMA-ScR) [12,13].

### Review questions

1) What volume of scientific literature exists in this area?
2) What aspects related to the nutritional quality of cell-cultured food products are covered by the research?
3) What are the knowledge gaps in this area?

Inclusion criteria

Publications identified during the search will be included in the review if they meet the following criteria:

• Scientific publications (peer-reviewed journal articles; reports from regulatory, scientific, or non-governmental bodies);
• Report or discuss nutritional considerations of cell-cultured food products derived from plant or animal cells; and
• Publications written in English or French.

Identified publications will be excluded if they:

• Are not a primary or secondary peer-reviewed scientific publication (e.g., editorials, letters to the editor, abstracts or collections of abstracts, theses);
• Are not a report, position statement, guideline, or scientific opinion published by a government, inter-governmental agency, or non-governmental organization (e.g., newspaper or popular magazines, webpages, blogs, or social media feeds, newsletters, speeches, slide decks);
• Exclusively discuss topics about isolated compounds (e.g., recombinant proteins, fatty acids) or oils;
• Exclusively reports or discusses non-nutritional issues; or
• Exclusively discuss foods that contain cultured cells from organisms other than plants or animals.

### Types of sources

This scoping review will consider scientific publications of any primary or secondary design published in peer-reviewed journals, as well as reports from regulatory, scientific, or non-governmental bodies. Relevant publications will be identified through searches in electronic databases and the grey literature.

For the search of bibliographic databases, an experienced research librarian developed and tested search strategies through an iterative process in consultation with the review team. An initial limited search was undertaken to identify articles on the topic. The text words contained in the titles and abstracts of relevant articles, and the index terms used to describe the articles were used to develop a full search strategy for Embase, Medline, Food Science and Technology Abstracts, Global Health, CAB Abstracts and Agricola (see Additional information). Only reports published in English or French will be included. No date limitation was applied. Supplemental Material Table 1 provides an example of the search strategy for the Embase database. Deduplicated search results will be uploaded to Covidence systematic review software for screening (Veritas Health Innovation, Melbourne, Australia. Available at www.covidence.org).

**Table 1:** Search Strategy utilized for the Embase database **Embase:** 1974 to 2024 January 22.

#	Searches	Results
1	cell culture/ and agriculture/	98
2	(artificial meat* or cellular agricultur* or cellular aquacultur* or cellular meat* or clean meat* or cultivated meat* or cultured meat* or culturing meat* or engineered meat*).tw,kw,kf.	468
3	((cell-based adj3 (beef* or food? or meat* or milk or pork or poultry or seafood* or sea food*)) or cell-based fish*).tw,kw,kf.	60
4	((cell-based or lab based or lab grown or lab made) adj3 (breastmilk or breast milk or human milk)).tw,kw,kf.	1
5	(cultured beef* or cultivated beef* or cultured burger* or cultivated burger* or cultured food* or cultivated food* or cultivated milk* or cultured pork* or cultivated pork* or cultured poultry* or cultivated poultry* or cultured protein* or cultured seafood* or cultivated seafood* or cultured sea food* or cultivated sea food* or in vitro beef* or in vitro food* or in vitro meat* or in vitro pork or in vitro poultry or in vitro seafood* or in vitro sea food* or synthetic meat* or synthetic seafood*).ti,kw,kf.	90
6	(cultured beef* or cultivated beef* or cultured burger* or cultivated burger* or cultured food* or cultivated food* or cultivated milk* or cultured pork* or cultivated pork* or cultured poultry* or cultivated poultry* or cultured protein* or cultured seafood* or cultivated seafood* or cultured sea food* or cultivated sea food* or in vitro beef* or in vitro food* or in vitro meat* or in vitro pork or in vitro poultry or in vitro seafood* or in vitro sea food* or synthetic meat* or synthetic seafood*).ab. /freq=2	38
7	((lab based or lab grown or factory grown) adj3 (beef* or fish or food? or meat* or milk or pork or poultry or seafood* or sea food*)).tw,kw,kf.	51
8	1 or 2 or 3 or 4 or 5 or 6 or 7	670
9	(conference abstract or editorial or letter or note).pt.	8103,564
10	8 not 9	549
11	limit 10 to (English or French)	535

For the grey literature search, the research librarian created effective search strings for Google and other relevant databases or search engines and compiled a list of relevant targeted websites. The search plan was optimized to minimize the risk of omitting relevant sources. Any relevant articles will be retained, stored, and de-duplicated by using a Microsoft Excel file.

### Evidence selection

Following the search, all identified databases records will be uploaded to Covidence systematic review software for screening (Veritas Health Innovation, Melbourne, Australia. Available at www.covidence.org). Following a pilot test, titles and abstracts will then be screened by two independent reviewers against the inclusion criteria for the review. Potentially relevant records will be retrieved in full and tracked through Covidence. The full text of selected citations will then be assessed in detail by two independent reviewers against the inclusion criteria. Reasons for exclusion of sources of evidence at the full text level that do not meet the inclusion criteria will be recorded and reported in the scoping review. Any disagreements between reviewers at each stage of the screening process will be resolved through discussion and consensus, or, if necessary, with an impartial additional reviewer.

All non-database (grey literature) records will be tracked using a Microsoft Excel spreadsheet. Abstracts, executive summaries, or table of contents (whichever is available) will be screened, followed by full-text screening.

The results of the search will be reported in full in the final scoping review and presented in a (PRISMA-ScR) flow diagram.

### Data extraction

Data will be extracted from the records included in the scoping review by one reviewer and verified by a second reviewer. The data extracted will focus on:

• Citation, Author(s)
• Study type (e.g., study design)
• Type of cellular agriculture product
• Topics covered related to nutrition
• Key findings that relate to the scoping review questions
• Knowledge gaps reported
• Declared sponsors or reported funding

Data extraction from the grey literature publications will include:

• Source/Organization
• Year of publication
• Key findings related to the scoping review questions
• Knowledge gaps reported
• Declared sponsors or reported funding (outside of the named organization, if applicable)

Data extraction will be standardized using tools developed by the reviewers. These tools will be piloted to assess the feasibility of the process for extracting data from each of the included records. Any modifications will be detailed in the full scoping review. Any disagreements between reviewers during data extraction will be resolved through discussion or with the involvement of a third reviewer. All extracted information will be compiled into detailed evidence tables in Microsoft Excel.

## Data analysis and presentation

The results of this scoping review will be described in detail. Tables and charts will be used to illustrate the findings, accompanied by a narrative summary explaining how they relate to the objectives of the review. The findings will inform and strengthen Health Canada’s nutritional safety assessments of novel foods derived from cellular agriculture. Identified research gaps will provide opportunities for future research in this area.

## Protocol validation

Not applicable.

## Limitations

While this scoping review protocol is comprehensive and follows established methodological guidelines, it has some limitations. Restricting the search to English and French publications may lead to language bias and the exclusion of relevant studies published in other languages. The inclusion criteria also exclude certain sources, such as theses, conference abstracts, and non-peer-reviewed materials, which may omit emerging evidence in this rapidly evolving field. Finally, as a scoping review, the study will summarize existing evidence but will not assess the quality of included studies, limiting its ability to draw conclusion about the strength or reliability of the evidence base.

## Novelty

To the best of our knowledge, this scoping review is the first to systematically identify and synthesize existing evidence on the nutritional aspects of foods derived from cellular agriculture, an area that has not yet been comprehensively reviewed. To date, most discussions on cell-based foods have focused on technological feasibility, consumer acceptance, and regulatory challenges, with limited attention to their nutritional quality and health implications. By focusing specifically on the nutritional aspect, this review addresses a critical gap in the literature and provides evidence to support regulatory assessments of these emerging foods. Integration of both peer-reviewed and grey literature in this study further ensures a comprehensive overview of current knowledge. The findings will inform nutritional safety assessments, identify priority research gaps, and guide future investigations to support the safe and responsible development of cell-cultured food products globally.

## Related research article

None.

## Supplementary material

Supplementary material and/or additional information [OPTIONAL]

## Author's Role

**Karima Benkhedda**: Conceptualization, Methodology, Validation, Supervision, Writing-Reviewing and Editing, Visualization. **Matthew Parrott**: Conceptualization, Methodology, Validation, Writing-Reviewing and Editing, Visualization. **Atiq Rehman**: Writing-Reviewing and Editing. **Subhadeep Chakrabarti:** Writing-Reviewing and Editing. **Stephanie K. Nishi**: Methodology, Validation, Writing-Reviewing and Editing, Visualisation. **Vincent C.H. Wong:** Methodology, Validation; Writing-Reviewing and Editing, Visualisation **Laura Kenney**: Methodology, Writing-Reviewing and Editing. **Chao-Wu Xiao**: Validation, Writing-Reviewing and Editing. **Mélanie Whiteside**: Writing-Reviewing and Editing.

## Declaration of competing interest

The authors declare that they have no known competing financial interests or personal relationships that could have appeared to influence the work reported in this paper.

## Data Availability

Data will be made available on request.
